# An *Abies procera*-derived tetracyclic triterpene containing a steroid-like nucleus core and a lactone side chain attenuates *in vitro* survival of both *Fasciola hepatica* and *Schistosoma mansoni*

**DOI:** 10.1016/j.ijpddr.2018.10.009

**Published:** 2018-10-26

**Authors:** Helen L. Whiteland, Anand Chakroborty, Josephine E. Forde-Thomas, Alessandra Crusco, Alan Cookson, Jackie Hollinshead, Caroline A. Fenn, Barbara Bartholomew, Peter A. Holdsworth, Maggie Fisher, Robert J. Nash, Karl F. Hoffmann

**Affiliations:** a The Institute of Biological, Environmental and Rural Sciences (IBERS), Aberystwyth University, SY23 3DA, Wales, UK; b PhytoQuest Limited, Plas Gogerddan, Aberystwyth, Ceredigion, SY23 3EB, Wales, UK; c Ridgeway Research Limited, Park Farm Buildings, Park Lane, St Briavels, Gloucestershire, GL15 6QX, England, UK

**Keywords:** *Abies procera*, *Abies grandis*, Triterpenoid, Anthelmintic drug discovery, Neglected tropical diseases, *Fasciola hepatica*, *Schistosoma mansoni*

## Abstract

Two economically and biomedically important platyhelminth species, *Fasciola hepatica* (liver fluke) and *Schistosoma mansoni* (blood fluke), are responsible for the neglected tropical diseases (NTDs) fasciolosis and schistosomiasis. Due to the absence of prophylactic vaccines, these NTDs are principally managed by the single class chemotherapies triclabendazole (*F. hepatica*) and praziquantel (*S. mansoni*). Unfortunately, liver fluke resistance to triclabendazole has been widely reported and blood fluke insensitivity/resistance to praziquantel has been observed in both laboratory settings as well as in endemic communities. Therefore, the identification of new anthelmintics is necessary for the sustainable control of these NTDs in both animal and human populations. Here, continuing our work with phytochemicals, we isolated ten triterpenoids from the mature bark of *Abies* species and assessed their anthelmintic activities against *F. hepatica* and *S. mansoni* larval and adult lifecycle stages. Full ^1^H and ^13^C NMR-mediated structural elucidation of the two most active triterpenoids revealed that a tetracyclic steroid-like nucleus core and a lactone side chain are associated with the observed anthelmintic effects. When compared to representative mammalian cell lines (MDBK and HepG2), the most potent triterpenoid (700015; anthelmintic EC_50_s range from 0.7 μM–15.6 μM) displayed anthelmintic selectivity (selectivity indices for *F. hepatica*: 13 for newly excysted juveniles, 46 for immature flukes, 2 for mature flukes; selectivity indices for *S. mansoni*: 14 for schistosomula, 9 for immature flukes, 4 for adult males and 3 for adult females) and induced severe disruption of surface membranes in both liver and blood flukes. *S. mansoni* egg production, a process responsible for pathology in schistosomiasis, was also severely inhibited by 700015. Together, our results describe the structural elucidation of a novel broad acting anthelmintic triterpenoid and support further investigations developing this compound into more potent analogues for the control of both fasciolosis and schistosomiasis.

## Highlights

• *Abies* species contain anthelmintic phytochemical triterpenoids.
• The triterpenoid 700015 affects larval, juvenile and adult fluke viabilities.
• 700015 is moderately selective against both fluke species.

## Graphical abstract

**Figure:**
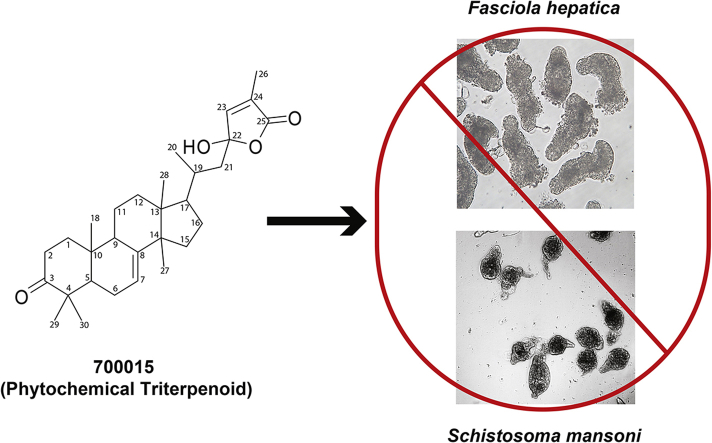

## 1 Introduction

The Neglected Tropical Disease (NTD) causing flatworms *Fasciola hepatica* (liver fluke responsible for fasciolosis) and *Schistosoma mansoni* (blood fluke responsible for schistosomiasis) are amongst some of the most successful parasites on the planet (Collins, 2017). Fasciolosis is predominantly a disease of cattle and sheep, where tissue damage induced by intra-definitive host parasite migration causes a loss of ∼ US $3.2 billion per annum (Kelley et al., 2016). However, human infections are also possible, with a recent estimate indicating that ∼50 million people are currently infected worldwide (Nyindo and Lukambagire, 2015). Morbidity associated with schistosomiasis is due to eggs, released by schistosome pairs into the definitive host's blood stream, inducing T helper 2 (Th2)-mediated inflammation upon being trapped in organs such as the liver, intestines and bladder (Pearce et al., 1992; Warren, 1978). The long-term consequences of chronic inflammation leads to disease development in more than 200 million people per annum, often killing thousands to hundreds of thousands in endemic areas every year (Hotez and Fenwick, 2009). When considering that both fasciolosis and schistosomiasis are often found co-endemic (Esteban et al., 2003; Yabe et al., 2008; Krauth et al., 2015), tremendous pressure in the health care budgets and One-Health objectives can develop in countries where resources are insufficient to meet competing financial demands. This situation is likely to be further exacerbated as the geographical ranges of both diseases expand (Fox et al., 2011; Holtfreter et al., 2014; Caminade et al., 2015; Hotez, 2018).

Current strategies for liver and blood fluke control are predominantly restricted to single class anthelmintics. As triclabendazole kills all stages of *Fasciola* development in the definitive host (newly excysted juveniles – NEJs, immature flukes and sexually-mature flukes), it has been the preferred drug of choice since its introduction (Kelley et al., 2016). However, overreliance on triclabendazole has led to overuse and the rapid development and spread of drug resistant liver flukes in both animals and humans (Brennan et al., 2007; Winkelhagen et al., 2012). Without a prophylactic vaccine, the development of new anthelmintics is urgently needed for the sustainable control of fasciolosis. A similar situation exists for schistosomiasis, where overuse of praziquantel threatens the rise of drug insensitive or resistant parasites in countries engaged in wide-scale, mass drug administration (MDA) programmes (Cupit and Cunningham, 2015). As there are also no anti-schistosomal vaccines currently registered for use and praziquantel is not effective against all stages of schistosome development in the definitive host (Wu et al., 2011), the identification of new anthelmintic drugs is a necessary component of sustainable schistosomiasis control beyond 2020 (WHO, 2012). Ideally, a single compound that displays dual activity against definitive host lifecycle stages (juveniles, immature and mature flukes) of both liver and blood flukes would be advantageous in contributing to the control of fasciolosis and schistosomiasis.

Towards this end, we previously have described the anthelmintic effects of a *Lycium chinense* derived diterpenoid, 7-keto-sempervirol, against both *F. hepatica* and *S. mansoni* juveniles and adults (Edwards et al., 2015). While these effects were moderate, follow-on studies detailed a total synthesis and medicinal chemistry optimisation approach to produce thirty 7-keto-sempervirol analogues; amongst these thirty were representative diterpenoids demonstrating increased anthelmintic potency and selectivity (Crusco et al., 2018). Here, broadening our search for structurally related phytochemicals, we report the anthelmintic activities of ten triterpenoids isolated from *Abies* sp. (Fir trees) on *F. hepatica* and *S. mansoni* parasites. Preliminary structural activity relationships (SAR) indicate that a tetracyclic steroid-like nucleus core and a lactone side chain are associated with the observed anthelmintic effects (e.g. reduced motility, altered phenotypes, decreased cellular proliferation, damaged surface, diminished egg production) of the most potent triterpenoids. These results extend the chemical space of plant-based natural products, which display anthelmintic activity against flukes of both veterinary and biomedical importance.

## 2 Materials and methods

### 2.1 Ethics statement

All procedures performed on mice (project license PPL 40/3700) and sheep (project licenses PPL 40/3593, P6D805744 and PA09B4E45) adhered to the United Kingdom Home Office Animals (Scientific Procedures) Act of 1986 as well as the European Union Animals Directive 2010/63/EU and were approved by Aberystwyth University's (AU) and Ridgeway Research Limited's (RRL) Animal Welfare and Ethical Review Bodies (AWERB).

### 2.2 Isolation of triterpenoids from abies species

A total of ten triterpenoid compounds were isolated from dichloromethane (DCM) extracts of the mature bark of *Abies* species grown in Wales (UK) by normal phase and reverse phase chromatographic systems. Photodiode array and mass spectrophotometry detection to monitor peak separations were also applied during the isolation process. For specific isolation of the most active compound 700015, mature branches (600 g) of *Abies procera* (the Noble Fir) were collected in January by Forest Research (now Natural Resources Wales) at Cefn Gethiniog, Talybont-on-Usk, Brecon, Powys (reference N10122) and given PhytoQuest code IG289; this starting material was used to purify 160 mg of pure (>95%) compound. Here, the DCM extraction of the freeze-dried ground material was conducted in a Soxhlett overnight and the extract cleaned using Diaion HP20 resin. Normal phase flash chromatography using Dionex cartridges and heptane:ethyl acetate (70:30) was monitored at 210 nm and the fraction further separated using preparative high performance liquid chromatography (HPLC, NovaPak08 radial compression) with water: acetonitrile: acetonitrile + 0.1% TFA (26:65:10) giving the pure compound. The second most active compound, 700234, was purified by the same method from 1 kg of mature bark of *Abies grandis* (the Grand Fir; collected in January from the same location) supplied by Natural Resources Wales (N10127), yielding 750 mg of pure (>95%) compound. A reference sample was given the PhytoQuest code IG294.

Compound 700015 gave a distinctive mass spectrum (EI, 70 eV) with major ion 325 atomic mass units (Waters Integrity) while 700234 had distinctive ions at 295 (100%) and 313 (50%) atomic mass units. The UV spectra were end absorbing (200 nm) and not informative. Using an analytical HPLC system, the retention time was 8.4 min for 700015 and 9.5 min for 700234. The analytical method used a C_8_ HPLC column (50 mm × 4.6 mm id x 3.5 μm, Waters) with a flow rate of 1.5 mL/min and a linear gradient that started at 90% water and 10% acetonitrile (containing 0.01% trifluoroacetic acid), rising to 100% acetonitrile over 6 min, which was held for a further 6 min. Structural elucidation was conducted using NMR spectroscopy on a 500 MHz Bruker Avance instrument. Mass spectrometry was performed on an Orbitrap Fusion Thermo Scientific with a Dionex UltiMate 3000 UHPLC system.

### 2.3 Structural elucidation of triterpenoid 700015

Colourless oil, structural elucidation involved two steps. Purified 700015 was characterised by high resolution mass spectrometry (HRMS) together with ^1^H, ^13^C and two-dimensional Nuclear Magnetic Resonance (NMR) spectroscopy. Peak lists for ^1^H NMR (500 MHz, CDCl_3_) δ/ppm: 6.83 (1H, broad s, 23), 5.43 (1H, m, 7), 2.41 (2H, m, 2) 2.00 (1H, m, 9), 1.87 (1H, m, 21) 1.86 (1H, m, 19), 1.81 (2H, m, 16), 1.75 (2H, m, 6), 1.68 (3H, s, 26), 1.65 (1H, m, 1), 1.61(1H, m, 11), 1.55 (1H, m, 1), 1.53 (1H, m, 11), 1.43 (1H, m, 21), 1.38 (2H, m, 15), 1.35 (1H, m, 5), 1.25 (3H, m, 12 and 17), 0.93 (6H, s, 29 and 30), 0.82 (6H, s, 20 and 27), 0.78 (3H, s, 18), 0.58 (3H, s, 28). Peak lists for ^13^C NMR (126 MHz, CDCl_3_) δ/ppm: 218.4 (C=O, 3), 171.4 (C=O, 25), 149.0 (C, 8), 148.9 (CH, 23), 131.8 (C, 24), 106.1 (C, 22), 53.9 (CH, 5), 52.7 (CH, 17), 52.7 (C, 14), 47.3 (C, 4), 45.9 (CH, 9), 44.0 (CH_2_, 21), 43.7 (C, 13), 36.2 (CH_2_, 15), 35.7 (C, 10), 35.7 (CH_2_, 1), 34.8 (CH_2_, 2), 33.3 (CH, 19), 33.3 (CH_2_, 12), 29.1 (CH_2_, 16), 28.4 (CH_3_, 30), 27.7 (CH_3_, 20), 23.5 (CH_2_, 11), 23.5 (CH_3_, 18), 23.4 (CH_3_, 27), 23.3 (CH_2_, 6), 22.7 (CH_3_, 29), 21.7 (CH_3_, 28) 10.3 (CH_3_, 26). HRMS-ESI m/z: [M + H]^+^ calculated for C_30_H_45_O_4_ is 469.3318 and experimentally determined to be 469.3317. According to SciFinder (https://www.cas.org/products/scifinder) searches, 700015 has been previously isolated from *Abies holophylla* (Kim et al., 2018) and *Abies sibirica* (Korolev et al., 2003; Handa et al., 2013).

### 2.4 Structural elucidation of triterpenoid 700234

Colourless oil, structural elucidation involved two steps. Purified 700234 was characterised by HRMS and two-dimensional (^1^H, ^13^C) NMR spectroscopy. Peak lists for ^1^H NMR (500 MHz, MeOD) δ/ppm: 6.91 (1H, m, 23), 5.68 (1H, m, 11), 5.55 (1H, m, 6), 3.42 (2H, m, 3 and 16), 2.29 (1H, m, 12), 2.09 (1H, m, 7), 2.08 (1H, m, 12), 2.07 (1H, m, 1), 2.04 (1H, m, 19), 1.94 (H, m, 17), 1.93 (1H, m, 21), 1.91 (1H, m, 15), 1.89 (3H, d, *J* = 7.5 Hz, 26), 1.87 (1H, m, 7), 1.86 (1H, m, 21), 1.82 (1H, m, 2), 1.56 (2H, m, 2 and 15), 1.36(1H, m, 1), 1.11 (3H, s, 18), 1.02 (3H, s, 29), 1.01 (3H, s, 30), 0.98 (6H, s, 27 and 28), 0.92 (3H, d, overlayed, 20). Peak lists for ^13^C NMR (126 MHz, MeOD) δ/ppm: 174.6 (C=O, 25), 157.8 (C, 5), 149.9 (CH, 23), 147.9 (C, 9), 132.3 (C, 24), 124.9 (CH, 6), 120.9 (CH, 11), 109.4 (C, 22), 78.2 (CH, 16), 75.2 (CH, 3), 51.7 (C, 4), 50.6 (C, 14), 49.9 (C, 13), 48.8 (CH_2_, 15), 39.8 (CH_2_, 21), 39.7 (C, 10), 37.2 (CH, 17), 36.9 (CH_2_, 7), 36.4 (CH, 8), 35.1 (CH_2_, 1), 30.3 (CH, 19), 27.5 (CH_3_, 30), 27.4 (CH_3_, 18), 24.9 (CH_2_, 2), 24.7 (CH_3_, 29), 19.8 (CH_3_, 27), 19.1 (CH_3_, 28), 18.9 (CH_3_, 20), 10.3 (CH_3_, 26). HRMS-ESI m/z: [M + H]^+^ calculated for C_30_H_43_O_4_ is 467.3161 and experimentally determined to be 467.3169. According to searches in PubChem (https://pubchem.ncbi.nlm.nih.gov/search/) and SciFinder (https://www.cas.org/products/scifinder), 700234 represents a novel chemical entity.

### 2.5 Compound storage and handling

All ten triterpenoids were solubilised in DMSO (Fisher Scientific, UK) to a stock concentration of 10 mM and stored at −20 °C until required. For all fluke screens, compounds were further diluted to a working concentration of 1.6 mM in DMSO. Positive controls for *S. mansoni* screens included praziquantel (Sigma-Aldrich, UK) and auranofin (Sigma-Aldrich, UK), which were also diluted in DMSO to a stock concentration of 10 mM and working concentration of 1.6 mM. A positive control for *F. hepatica* screens included triclabendazole (Sigma-Aldrich, UK), which was diluted in DMSO to a working concentration of 10 mM.

### 2.6 Screening of *F. hepatica* newly excysted juveniles (NEJs)

Metacercariae of a *F. hepatica* Italian strain were supplied by RRL and an excystment was performed as previously described (Crusco et al., 2018). After excystment, NEJs were distributed into a 24 well tissue culture plate at a density of 25 parasites per well containing 1 mL of fresh RPMI 1640 media (Gibco, Paisley, UK) supplemented with 1% v/v Foetal Calf Serum (Gibco, Paisley, UK) and 1X v/v antibiotic/antimycotic solution (Sigma-Aldrich, UK). All ten triterpenoids were added to respective wells and NEJ/compound co-cultures were incubated at 37 °C in an atmosphere containing 5% CO_2_ for 72 h at a final concentration of 10 μM; phenotype and motility was independently scored using the scoring matrix as described previously (Edwards et al., 2015). Controls included NEJs cultured in 0.1% DMSO (negative) or 10 μM Triclabendazole (in 0.1% DMSO; positive). A dose response titration of 700015 involved the co-cultivation (as described above) of 25 NEJs per well containing final triterpenoid concentrations of 10 μM, 5 μM, 2.5 μM, 1.25 μM and 0.625 μM (all in 0.1% DMSO).

### 2.7 Screening of immature and adult *F. hepatica* liver flukes

Lambs (Texel Mule X, 6 months old) were orally infected with 200 F*. hepatica* (Italian strain) metacercariae and, four and 8 wk later, immature and mature liver fluke were obtained and prepared as previously described (Crusco et al., 2018). Immature flukes (n = 3/condition) were transferred to 6-well plates (Thermo Scientific, Denmark) containing 3 mL of RPMI 1640 media (Gibco, Paisley, UK) supplemented with 2.5% HEPES (Sigma-Aldrich), 1X v/v antibiotic/antimycotic solution (Gibco, Paisley, UK) and 1% Foetal Bovine Serum (Gibco, Paisley, UK). Immature parasites were co-cultured at 37 °C in an atmosphere containing 5% CO_2_ with 700015 at 40 μM, 13.3 μM and 4.4 μM for 72 h; compound-induced motility defects were scored as previously described (Crusco et al., 2018).

Adult liver flukes (n = 3/condition) were transferred to 15 mL falcon tubes containing 6 mL of the same medium used for cultivating immature flukes and co-cultured at 37 °C in an atmosphere containing 5% CO_2_ with 700015 at 40 μM, 13.3 μM and 4.4 μM for 72 h. Every 24 h, 2 mL of fresh media and 700015 was added to each of the cultures; compound-induced motility defects were again scored according to Crusco et al. (2018). In both 4 wk old and 8 wk old liver fluke screens, control parasites included those treated with 0.4% DMSO (negative) or 40 μM Triclabendazole (in 0.4% DMSO, positive).

### 2.8 Screening of *S. mansoni* schistosomula

*Biomphalaria glabrata* (NMRI strain) snails infected with *S. mansoni* (Puerto Rican strain) were shed for 2 h under light conditions at 26 °C. Cercariae were collected, mechanically transformed into schistosomula (Colley and Wikel, 1974) and subsequently prepared for high throughput screening (HTS) on the Roboworm platform as previously described (Crusco et al., 2018; Nur et al., 2017). Schistosomula were added to each well containing triterpenoids (10 μM in 0.625% DMSO) at a density of 120 parasites per well. Parasites were cultured at 37 °C in an atmosphere containing 5% CO_2_ for 72 h with phenotype and motility of each parasite quantified using a previously described image analysis model (Paveley et al., 2012). Control treatments included schistosomula cultured in the presence of praziquantel (10 μM in 0.625% DMSO), auranofin (10 μM in 0.625% DMSO) and DMSO (0.625%). A dose response titration of 700015 involved the co-cultivation (as described above) of 120 schistosomula per well containing final triterpenoid concentrations of 10 μM, 5 μM, 2.5 μM, 1.25 μM and 0.625 μM (all in 0.625% DMSO).

### 2.9 Screening of juvenile *S. mansoni* blood flukes (3-week worms)

Juvenile *S. mansoni* adult parasites were recovered from MF-1 mice (Harlan, UK) that were infected percutaneously three weeks earlier with 4000 cercariae. Worms were transferred into 50 mL falcon tubes and centrifuged at 300×*g* for 2 min. This pellet was collected, washed in phenol-red free DMEM and subjected to repeat centrifugation. This procedure was repeated a further two times, where on the final wash, the parasites were pelleted by gravity. This final, washed pellet was collected and placed in culture media (DMEM media (Gibco, Paisley, UK) supplemented with 10% v/v Hepes (Sigma-Aldrich, Gillingham, UK), 10% v/v Foetal Bovine Serum (Gibco, Paisley, UK), 0.7% v/v 200 mM L-Glutamine (Gibco, Paisley, UK) and 1X v/v penicillin-streptomycin (Gibco, Paisley, UK).

A total of 6–19 juvenile worms were transferred to each well of a 96 well plate containing a final volume of 200  μL of media containing 700015 at the following concentrations: 15 μM, 7.5 μM, 3.75 μM and 1.83 μM (in 1.25% DMSO). This was repeated three times (n = 3). Parasites were cultured at 37 °C in an atmosphere containing 5% CO_2_ for 72 h at which time worm motility was scored between 0 and 4: 0 = dead, 1 = movement of the suckers only and slight contraction of the body, 2 = movement at the anterior and posterior regions only, 3 = full body movement but sluggish and 4 = normal movement.

### 2.10 Screening of adult *S. mansoni* blood flukes (7-week worms)

Adult *S. mansoni* parasites were recovered by hepatic portal vein perfusion from MF-1 mice (Harlan, UK) that were percutaneously infected seven weeks earlier with 200 cercariae. Three adult worm pairs per well, in duplicate, were transferred into 48 well plates (Fisher Scientific, Loughborough, UK) and cultured at 37 °C in an atmosphere containing 5% CO_2_ in DMEM media (Gibco, Paisley, UK) containing 10% v/v HEPES, 10% v/v Foetal Bovine Serum, 0.7% v/v 200 mM L-Glutamine and 1X penicillin-streptomycin (Gibco, Paisley, UK). A total of 3 biological repeats were conducted giving a total of 18 males and 18 females dosed per treatment. Worms were dosed with test compounds at 20 μM, 10 μM, 5 μM, 2.5 μM, 1.25 μM and 0.625 μM (in 1.25% DMSO) for 72 h. Adult worms were scored manually at 72 h using the WHO-TDR metric scoring system as described previously (Ramirez et al., 2007). At 72 h, the medium from each well was collected, centrifuged at 1000 rpm for 2 min, supernatant removed and remaining egg pellet re-suspended in 10% v/v formalin. Normal shaped eggs that were oval and contained a fully-formed lateral spine were subsequently counted.

### 2.11 Preparation of adult *F. hepatica* and *S. mansoni* worms for scanning electron microscopy (SEM)

Adult liver and blood flukes were cultivated (as described above) in sub lethal concentrations of 700015 (*F. hepatica* – 13.3 μM; *S. mansoni* – 10 μM) for 72 h. Afterwards, flukes were prepared for SEM as previously described (Crusco et al., 2018).

### 2.12 Quantification of EdU positive cells in adult *S. mansoni* worms

Adult *S. mansoni* worms were cultured (as described above) for 72 h in a sub lethal concentration of 700015 (10 μM). After 72 h, a 1  μL aliquot of 10 mM EdU was added to the culture media and incubated for a further 24 h. Worms were subsequently collected and fixed as described previously (Collins et al., 2013; Geyer et al., 2018). Anterior regions of both sexes were imaged on a Leica TCS SP5II confocal microscope using a 40× lens. A total of 150 Z-stacks were obtained for each individual worm (n = 10 for males, n = 5–7 females). EdU positive cells were quantified using Imaris v8.2 (BitPlane, UK) by analysing the fluorescent intensity of DAPI and EdU expressed as a total volume (μm^3^) occupied by each fluorophore.

### 2.13 MTT assay on HepG2 and madin darby bovine kidney (MDBK) cells

Overt cytotoxicity of 700015 was assessed on both human HepG2 and bovine MDBK (NBL-1) cells as described previously (Crusco et al., 2018). Briefly, 2 × 10^4^ cells/well (HepG2) or 7.5 × 10^3^ cells/well (MDBK) were seeded in black walled 96-well microtiter plates (Fisher Scientific, Loughborough, UK) and incubated for 24 h at 37 °C in a humidified atmosphere containing 5% CO_2_. To each well, 700015 was subsequently added to obtain final concentrations (in 1% DMSO) of 100 μM, 75 μM, 50 μM, 25 μM, 10 μM and 5 μM (HepG2 cells) or 100 μM, 75 μM, 50 μM, 20 μM, 10 μM and 5 μM (MDBK cells); negative (1% DMSO) and positive (1% v/v Triton X-100) control wells were included for both cell types. Following a further incubation for 24 h, the MTT assay was performed as previously described (Nur et al., 2017; Crusco et al., 2018).

### 2.14 Statistics

All Statistical analyses were conducted using GraphPad Prism 7 software. To determine significant differences amongst population means, a Kruskal-Wallis ANOVA followed by Dunn's multiple comparisons test was used.

## 3 Results and discussion

Investment in drug discovery research for NTDs is disproportionately low when compared to the impact that these diseases have on both animal and human lives. Therefore, pragmatic approaches for identifying new compounds to be used in *de novo* NTD drug discovery programmes have been developed and include drug repurposing/repositioning, drug rescuing, target repositioning and lead repurposing (Renslo and McKerrow, 2006; Panic et al., 2014; Klug et al., 2016; Gouveia et al., 2018). These approaches, often aided by public-private collaborations (Woods and Knauer, 2010; Ramamoorthi et al., 2014), can dramatically save time and money as the systematic optimisation of candidate molecules are minimised at this point in the pipeline.

In this regard, terpene/terpenoid phytochemicals have previously been explored and repurposed for their synergistic anti-parasitic capabilities; a well-known example is artemisinin, an endo-peroxide containing sesquiterpenoid lactone derived from *Artemisia annua*. Originally reported to inhibit *Plasmodium* proliferation (Su and Miller, 2015), artemisinin has subsequently been shown to affect other parasitic protozoan and helminth species (Loo et al., 2017; Lam et al., 2018). Of interest to the current study, *in vitro* cultivated *F. hepatica* and *S. mansoni* are both susceptible to artemisinin-based chemotherapies (Keiser and Utzinger, 2007). While the clinical progression of this particular sesquiterpenoid for both fasciolosis and schistosomiasis control has slowed (Hien et al., 2008; Utzinger et al., 2010), the search for other related di- or triterpenoids with anthelmintic activity continues (Kayser et al., 2003; Acevedo et al., 2017). Here, expanding on our previous investigations (Edwards et al., 2015; Crusco et al., 2018), we investigated the dual (*F. hepatica* and *S. mansoni*) anthelmintic activity of ten triterpenoids isolated from the bark of an indigenous coniferous species from Wales, United Kingdom (*A. procera* and *A. grandis*).

**Fig. 1:**
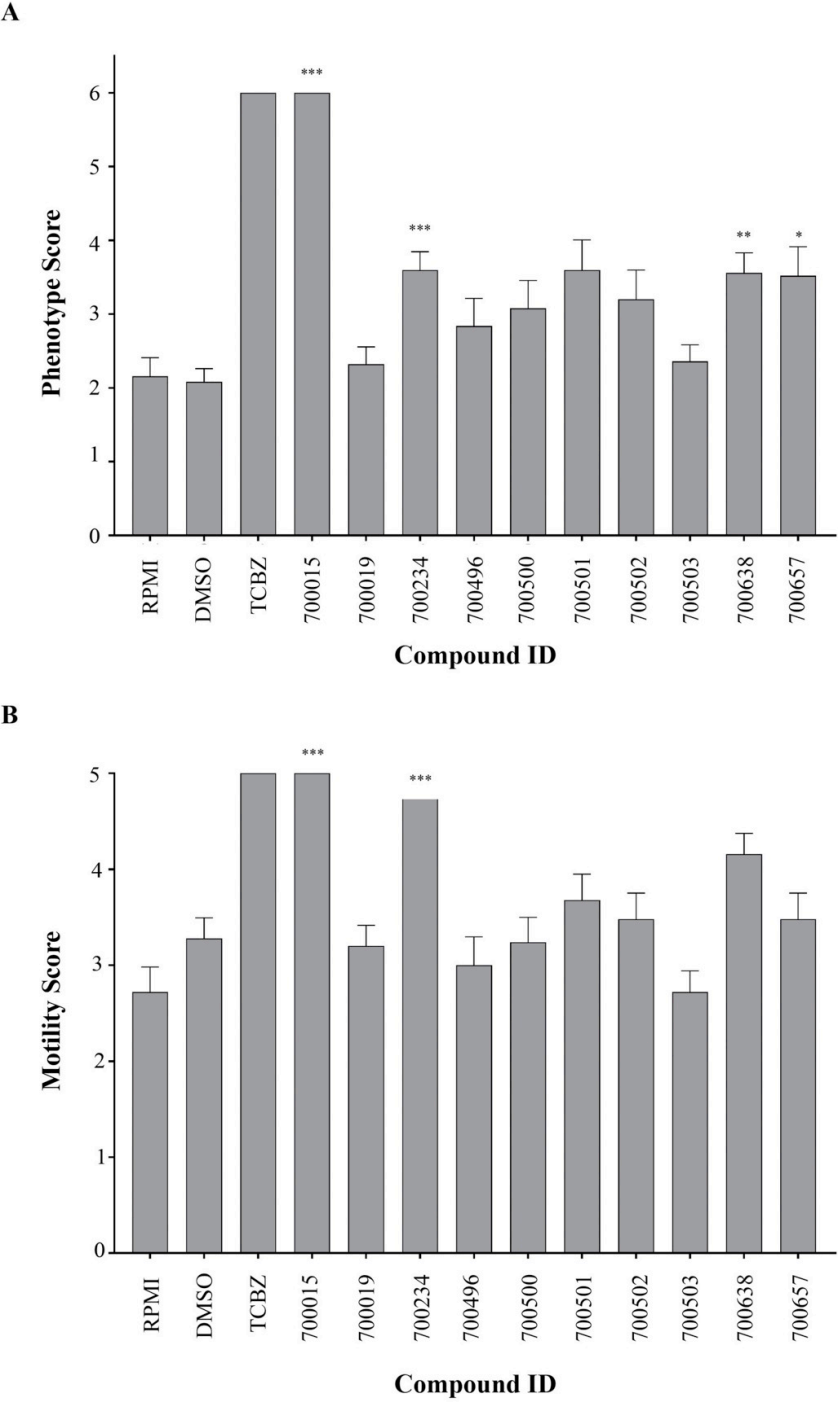
**Of the ten triterpenoids isolated from *Abies* sp., two (700015 and 700234) had significant activity against *Fasciola hepatica* newly excysted juveniles (NEJs)**. *F. hepatica* NEJs (25 per well) were incubated with ten triterpenoids at a concentration of 10 μM (in 0.1% DMSO) and cultured for 72 h at 37 °C in an atmosphere containing 5% CO_2_. Control wells (25 NEJs per well) included those containing 0.1% DMSO (negative) or 10 μM Triclabendazole (0.1% DMSO, positive). At 72 h, all NEJs were scored for phenotype **(A)** and motility **(B)** metrics as previously described (Edwards et al., 2015). *p = 0.05, **p = 0.01, ***p = 0.001.

**Fig. 2:**
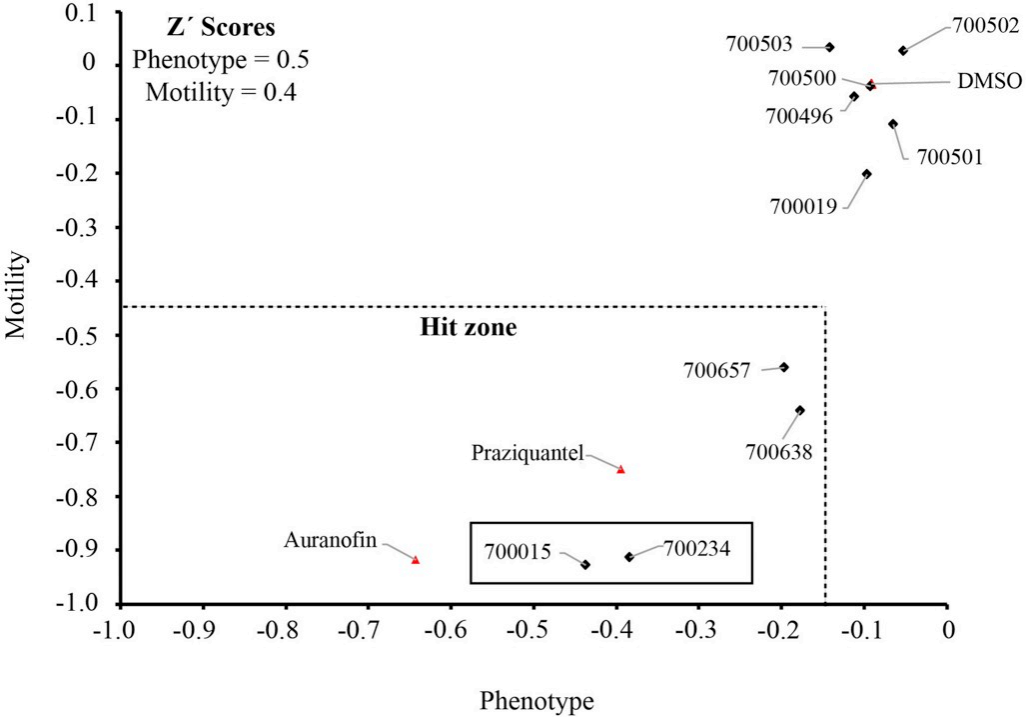
**Four triterpenoids, including 700015 and 700234, negatively affect *Schistosoma mansoni* schistosomula phenotype and motility metrics**. Mechanically transformed schistosomula (120 per well) were cultivated in the presence of triterpenoids (10 μM in 0.625% DMSO) for 72 h at 37 °C in an atmosphere containing 5% CO_2_. Control wells included schistosomula cultivated in DMSO (0.625%, negative), auranofin (in 0.625% DMSO, positive) and praziquantel (in 0.625% DMSO, positive). At 72 h, all schistosomula were scored for phenotype and motility as previously described (Paveley et al., 2012). Compounds were considered a hit (within the hit zone) when schistosomula phenotype fell below −0.15 and schistosomula motility fell below −0.35. The Z' values for this screen was 0.4 for motility and 0.5 for phenotype.

**Fig. 3:**
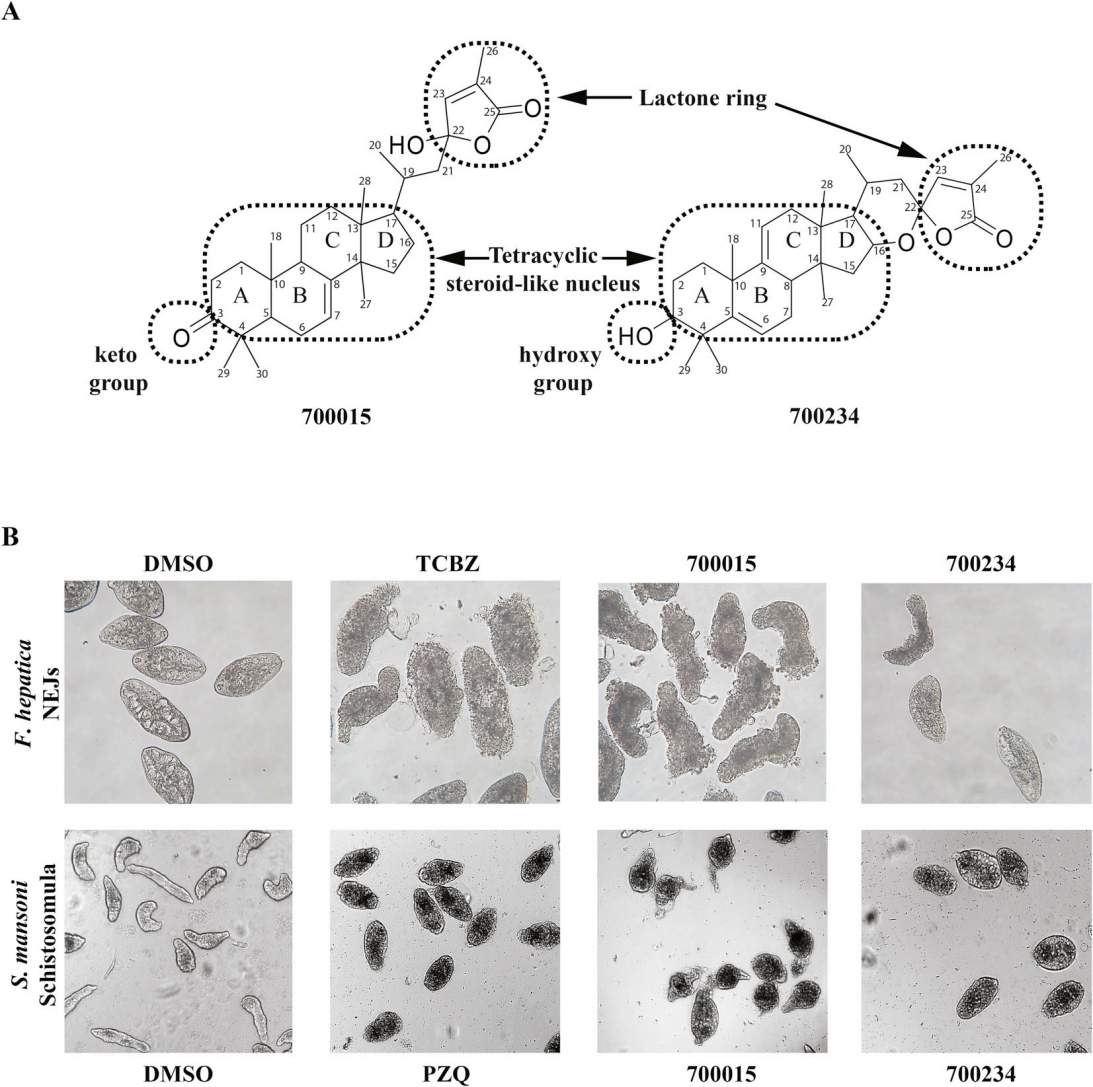
**A lactone ring is associated with the dual anthelmintic activity of the two most effective triterpenoids**. **(A)** The structural elucidation of triterpenoids 700015 and 700234 revealed a tetracyclic steroid-like nucleus core, a keto- (700015) or hydroxyl- (700234) group and a conserved lactone ring. ‘A, B, C, D’ represent the classic lettering system for steroids (Moss, 1989). Carbon numbering is derived from the ^13^C NMR structural elucidation of each triterpenoid. **(B)** Representative images of *F. hepatica* NEJs co-cultivated with DMSO (0.1%, negative control), TCBZ (10 μM in 0.1% DMSO, positive control), 700015 (10 μM in 0.1% DMSO) and 700234 (10 μM in 0.1% DMSO) obtained by bright field microscopy (20× objective) compared to representative images of *S. mansoni* schistosomula co-cultivated with DMSO (0.625%, negative control), PZQ (10 μM in 0.625% DMSO, positive control), 700015 (10 μM in 0.625% DMSO) and 700234 (10 μM in 0.625% DMSO) obtained by the high content imaging platform (10× objective). All parasite images were acquired at 72 h post parasite/compound co-cultivation.

Using a numerical matrix to quantify both phenotype and motility, the ten isolated triterpenoids (Supplementary Figure 1) were first tested (at 10 μM) for activity against the NEJ stage of *F. hepatica* (Fig. 1). Separate wells containing NEJs co-cultivated with triclabendazole (10 μM) or DMSO were also included as controls. Most of the triterpenoids tested did not significantly affect NEJ phenotype (60%, Fig. 1A) or motility (80%, Fig. 1B) after 72 h of co-culture. However, amongst the four compounds that significantly affected NEJ phenotype (700015, 700234, 700638 and 700657), two (700015 and 700234) also significantly affected NEJ motility. When compared to the positive control triclabendazole (10  μM), 700015 (>700234) was equally potent in affecting both NEJ phenotypic and motility metrics. In a second bioassay using an automated high throughput anthelmintic platform (Nur et al., 2017), these same four triterpenoids (at 10 μM) were also found to affect both *S. mansoni* schistosomula motility and phenotypic measures (Fig. 2, Hit zone). Amongst these four, 700015 and 700234, again, were the most effective (700015 > 700234 due to its position within the hit zone being closer to the origin) with anti-schistosomula activity after 72 h of co-culture being greater than that measured for praziquantel (10 μM). These results prompted us to investigate structural activity relationships (SAR) of the ten triterpenoids to provide an explanation underlying their differential effects on both *F. hepatica* and *S. mansoni* larval developmental stages (Fig. 3 and Supplementary Figure 1).

Amongst the ten triterpenoids tested (Supplementary Figure 1), five of them are considered pentacyclic triterpenoid saponins (700496, 700500, 700501, 700502, 700503). Interestingly, none of these five showed significant activity against either *F. hepatica* NEJs or *S. mansoni* schistosomula at the concentration tested (Table 1). However, when the glycoside moieties were absent or replaced (e.g. 700638), but the same pentacyclic structure of the saponin aglycone remained, the activity increased. We concluded from this preliminary SAR analysis that the presence of glycoside moieties decreased anthelmintic activity; this triterpenoid modification may be associated with decreased lipophilia (negative LogP values for the glycoside containing pentacyclic triterpenoids 700496, 700500, 700501, 700502 and 700503 are found when compared to the other triterpenoids in this study, Supplementary Figure 1) and the inability to cross heptalaminate membranes of both liver and blood flukes. However, as saponins are known to be hydrolysed when ingested (Francis et al., 2002), the *in vivo* anthelmintic characteristics could be quite different to those obtained from *in vitro* studies.

**Table 1:** Triterpenoid activity against *F. hepatica* newly excysted juveniles (NEJs) and *S. mansoni* schistosomula.

Triterpenoid	*F. hepatica*		*S.mansoni*	
	Phenotype	Motility	Phenotype	Motility
700015	✓	✓	✓	✓
700019	–	–	–	–
700234	✓	✓	✓	✓
700496	–	–	–	–
700500	–	–	–	–
700501	–	–	–	–
700502	–	–	–	–
700503	–	–	–	–
700638	✓	–	✓	✓
700657	–	–	✓	✓

Parasites were incubated for 72 h at 37 °C in a humidified atmosphere in appropriate media containing 10 μM triterpenoid and scored according to Materials and Methods. Triterpenoids not affecting (−) or affecting (✓) parasite phenotype and motility metrics are indicated.

More interesting is the structural comparison of the most active triterpenoids (700015 and 700234) to the less active triterpenoid (700657) and finally to the completely inactive triterpenoid 700019; all four compounds share a tetracyclic steroid-like nucleus (Fig. 3A and Supplementary Figure 1). While the substitution of a keto or hydroxy group on ring A (considering the classic triterpenoid lettering system (Moss, 1989)) does not affect anthelmintic activity (700234 and 700019 have a hydroxy group, while 700015 and 700657 contain a keto group), the lactone side chain of ring D could potentially have a major role (Fig. 3B). Indeed, both 700015 and 700234 share this feature (Fig. 3A) whereas 700019 has lost it (i.e. lactone ring has opened). Moreover, the greater activity of 700015 over 700234 could be explained by the flexibility of the lactone ring (i.e. 700015 has a more flexible lactone ring when compared to 7000234). The screening of additional triterpenoids with similar features could potentially confirm these preliminary SAR observations and provide important indications on how the anthelmintic activity of these molecules can be increased. Nevertheless, as 700015 displayed the greatest dual anthelmintic activity on NEJs and schistosomula (at 10 μM), we subsequently quantified the potency (dose response titrations) and selectivity (bovine and human cell line cytotoxicity) of this triterpenoid.

**Fig. 4:**
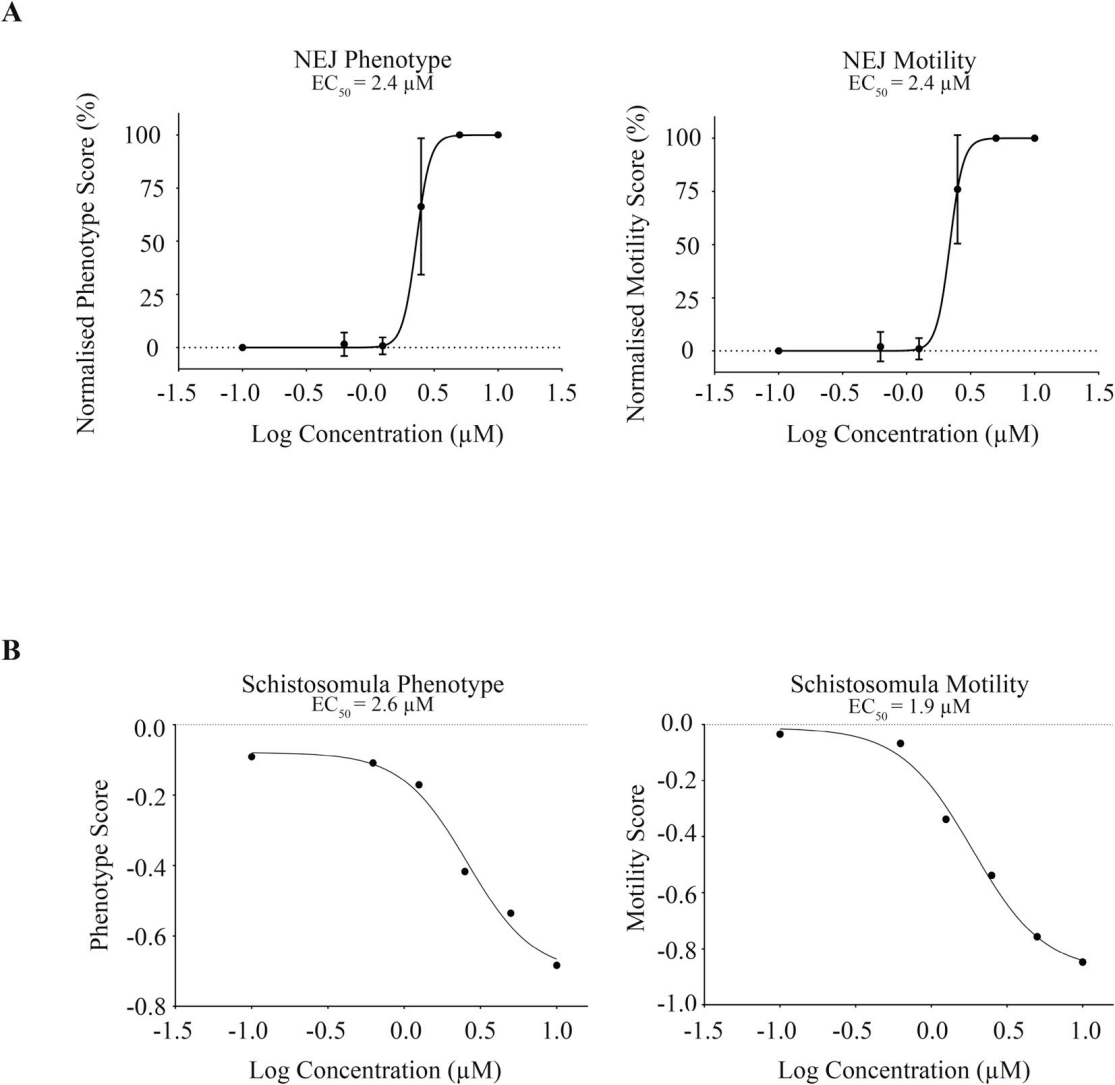
**Dose response titration of the most active triterpenoid on *Fasciola hepatica* newly excysted juveniles (NEJs) and *Schistosoma mansoni* schistosomula**. **(A)***F. hepatica* NEJs (25 per well) were incubated with 700015 at concentrations of 10 μM, 5 μM, 2.5 μM, 1.25 μM and 0.625 μM (in 0.1% DMSO) and cultured for 72 h at 37 °C in an atmosphere containing 5% CO_2_. A negative control well (25 NEJs per well) was included (0.1% DMSO) and is represented by −1.0 on the graphs. At 72 h, all NEJs were scored for phenotype and motility metrics. **(B)** Mechanically transformed schistosomula (120 per well) were cultivated in the presence of 700015 (10 μM, 5 μM, 2.5 μM, 1.25 μM and 0.625 μM in 0.625% DMSO) for 72 h at 37 °C in an atmosphere containing 5% CO_2_. A control well included schistosomula cultivated in DMSO (0.625%, negative; represented by −1.0 on the graphs). At 72 h, all schistosomula were scored for phenotype and motility. The Z' values for this screen was 0.4 for motility and 0.5 for phenotype.

**Fig. 5:**
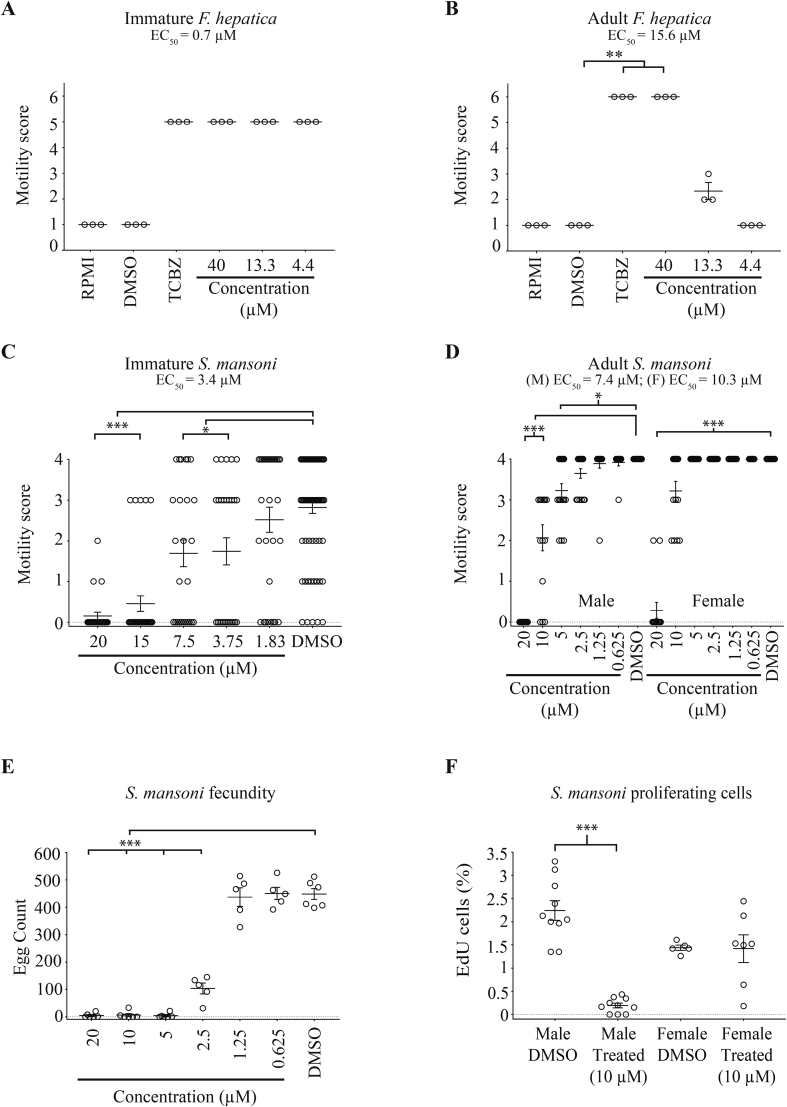
**Immature and adult stages of *Fasciola hepatica* and *Schistosoma mansoni* are affected by the most active triterpenoid 700015**. **(A)** Immature *F. hepatica* parasites (4 weeks post infection; n = 3) were cultured in the presence of a decreasing concentration of 700015 (40 μM, 13.3 μM and 4.4 μM in 0.4% DMSO) and motility scored at 72 h. Control parasites (n = 3 per condition) included those co-cultivated in RPMI only, RPMI containing 0.4% DMSO (negative) and TCBZ (40 μM in 0.4% DMSO, positive). **(B)** Adult *F. hepatica* parasites (8 weeks post infection; n = 3) were also cultured in the same 700015 titrations and motility metrics scored at 72 h and compared to control parasites. **(C)** Juvenile *S. mansoni* worms (3 weeks post infection; total number of parasites tested per condition = 26 - 33) were cultured in a titration of 700015 (20 μM, 15 μM, 7.5 μM, 3.75 μM and 1.88 μM in 1.25% DMSO) and motility scored at 72 h. Control parasites (n = 78) included those co-cultivated in the presence of 1.25% DMSO. **(D)** Adult *S. mansoni* worm pairs (7 weeks post infection; n = 18) were cultured in a decreasing dose of 700015 (20 μM, 10 μM, 5 μM, 2.5 μM, 1.25 μM and 0.625 μM in 0.2% DMSO) and motility scored at 72 h. **(E)** Media from adult *S. mansoni* worm cultures were collected at 72 h and counted for the presence of eggs. **(F)** Adult *S. mansoni* worm pairs were co-cultivated with a sub-lethal concentration of 700015 (10 μM) for 72 h and EdU positive cells quantified from the anterior (to ovaries and testes) region of each gender (male = 9–10 individuals; female = 5–7 individuals). In all panels, *p = 0.05, **p = 0.01, ***p = 0.001.

Dose response titrations of 700015 revealed similar potencies on both *F. hepatica* NEJs and *S. mansoni* schistosomula motility and phenotype metrics after 72 h of co-culture (Fig. 4). Here, EC_50_ values for *F. hepatica* NEJs were 2.4 μM (for both phenotype and motility) (Figs. 4A) and 1.9 μM (motility) to 2.6 μM (phenotype) for *S. mansoni* schistosomula (Fig. 4B). As these anthelmintic potency values exceeded our previous results obtained from related diterpenoids (Edwards et al., 2015; Crusco et al., 2018), we extended dose response titrations of 700015 against immature and mature fluke developmental stages of both species (Fig. 5).

In terms of *F. hepatica*, all concentrations of 700015 tested (40 μM, 13.3 μM and 4.4. μM) considerably affected the motility of immature (4 wk old) parasites (Fig. 5A); this triterpenoid-induced effect (EC_50_ = 0.7 μM) was equivalent to that seen for immature flukes co-cultured with triclabendazole (40 μM). Due to the limitations in immature fluke numbers, we were unable to titrate 700015 further and, thus, this EC_50_ is an estimate only. Adult (8 wk old) liver flukes were only significantly affected by higher concentrations of 700015 (40 μM). While this triterpenoid-induced effect (EC_50_ = 15.6 μM) was equivalent to that seen in adult flukes co-cultivated with triclabendazole (40 μM), a steep drop in activity was seen at 700015 concentrations below 13.3 μM. Nevertheless, as triclabendazole represents the only liver flukicide on the market with activity against NEJs, immature flukes and adults (Kelley et al., 2016), the results of these *in vitro* studies suggest that 700015 contains an important criterion for fasciolosis control considerations.

Regarding *S. mansoni*, 700015 significantly affected the motility of immature (3 wk old) parasites at all concentrations tested except 1.83 μM (Fig. 5C). While some individual variability was observed at each compound concentration, an EC_50_ of 3.4 μM was calculated for these mixed-sex parasite populations. Extending these assays to adult schistosome (7 wk old) mixed-sex cultures revealed a greater triterpenoid-mediated effect on male (EC_50_ = 7.4 μM) compared to female (EC_50_ = 10.3 μM) parasites. Although this gender biased (male > female) effect has been observed before for other (di- and sesqui-) terpenes/terpenoids (Edwards et al., 2015; de Oliveira et al., 2017), this is not always seen (de Moraes et al., 2014) and is most likely related to structural differences amongst this diverse class of compounds (de Moraes, 2015; Mafud et al., 2016). Interestingly, egg production was also significantly inhibited in mixed-sex adult worm cultures even at concentrations of 700015 below the EC_50_s (5 μM - 2.5 μM) calculated for this lifecycle stage (Fig. 5E). This decrease in egg production was directly correlated with a loss of male/female pairing. In contrast to the DMSO controls, where between 10 and 12 schistosome pairs (starting from n = 18 pairs) remained coupled after 72 h of culture, only 1–4 schistosome pairs (starting from n = 18 pairs) remained together when cultivated in the presence of 700015 (regardless of concentration). These results suggested that 700015 induced a stress response in adult schistosomes, which significantly impacted upon pairing and oviposition. While the molecular nature of this 700015-mediated stress response is currently unknown, it appears to also affect somatic stem cell (neoblast) proliferation (males > females) (Fig. 5F). As stem cell proliferation/differentiation and egg production are critical processes responsible for lifecycle transmission and immunopathology development (Pearce et al., 1992; Collins et al., 2013; Wang et al., 2018), these findings could hold relevance to schistosomiasis control. Due to limitations in parasite material, the effect of 700015 on *F. hepatica* stem cell proliferation and egg production was not examined in this study.

**Fig. 6:**
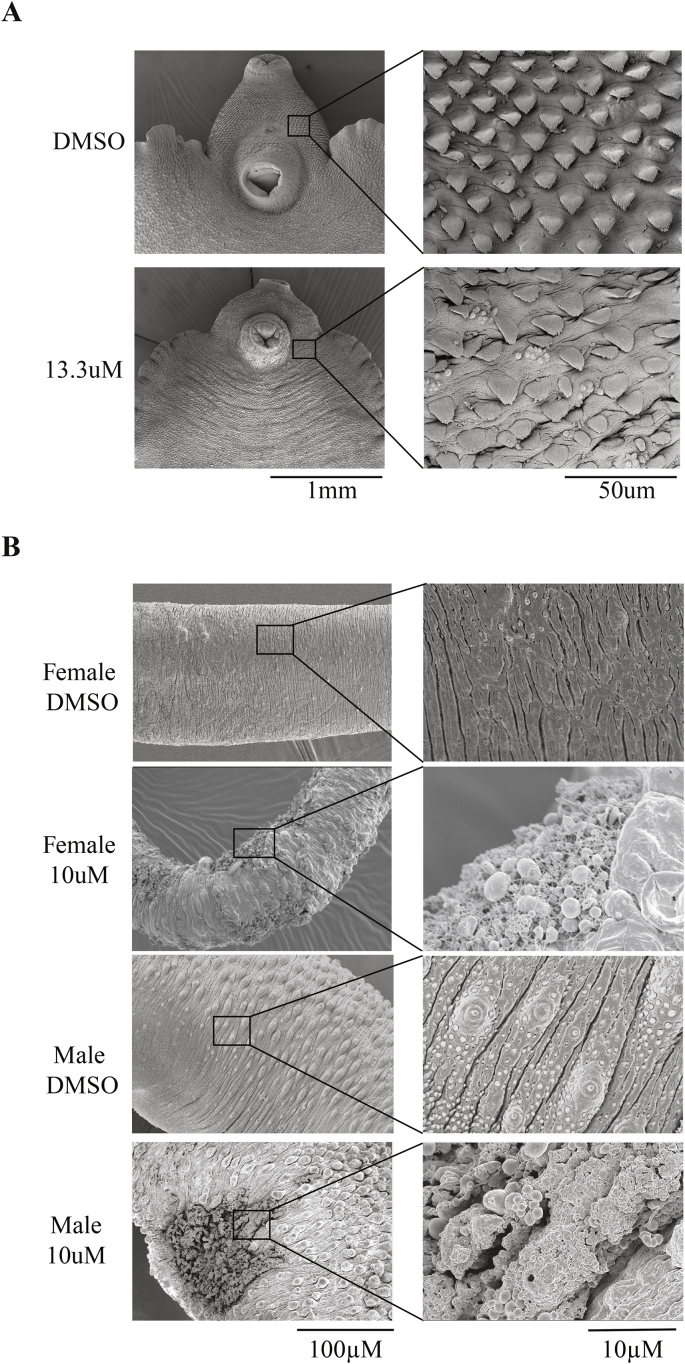
**The triterpenoid 700015 induces surface damage to both hermaphroditic *F. hepatica* and dioecious *S. mansoni* adults**. **(A)** Adult *F. hepatica* (n = 3) were cultured for 72 h in media containing a sub-lethal concentration (13.3  μM in 0.13% DMSO) of 700015. When compared to control parasites (0.13% DMSO; n = 3) at 72 h, 700015 induced spine erosion and irregular invaginations of the surface tegumental membranes surrounding the acetabulum. **(B)** Adult *S. mansoni* worm pairs (males and females; n = 7 pairs) were cultured for 72 h in a sub-lethal concentration (10 μM in 1.25% DMSO) of 700015. When compared to control parasites (1.25% DMSO; n = 7 pairs), 700015 led to tegumental surface disruption and membrane blebbing in 5/7 males and 5/6 females (1 female did not survive the SEM processing).

Previous studies have demonstrated that the anthelmintic activity observed for mono-, di- and sesqui-terpenes/terpenoids is correlated to surface damage of both *F. hepatica* and *S. mansoni* (Keiser and Utzinger, 2007; Keiser and Morson, 2008; de Moraes et al., 2013; Moraes et al., 2013; Edwards et al., 2015; de Oliveira et al., 2017; Crusco et al., 2018). Therefore, we next investigated whether tegumental surface membranes of both liver and blood flukes were negatively affected by sub-lethal concentrations (13.3 μM for *F. hepatica* adults, 10 μM for *S. mansoni* adults) of 700015 (Fig. 6). After 72 h of co-culture, clear alterations in the normal architecture of tegumental surfaces were observed in both species. For example, 700015 induced erosion of spines surrounding the acetabulum of liver flukes and mediated widespread tegumental dehydration/invagination (Fig. 6A). Small membranous blebs of the surface were also more apparent in 700015 treated liver flukes when compared to controls (DMSO). In blood flukes, 700015 led to severe disruption of the tegumental barrier with numerous membranous blebs and abrasions observed throughout the length of both male (5 out of 7 examined) and female (5 out of 6 examined) worms (Fig. 6B). As schistosome egg production is significantly affected at this triterpenoid concentration (Fig. 5E), it would appear that an intact tegumental barrier is partially responsible for maintaining this key parasitological trait.

**Supplementary Fig. 2:**
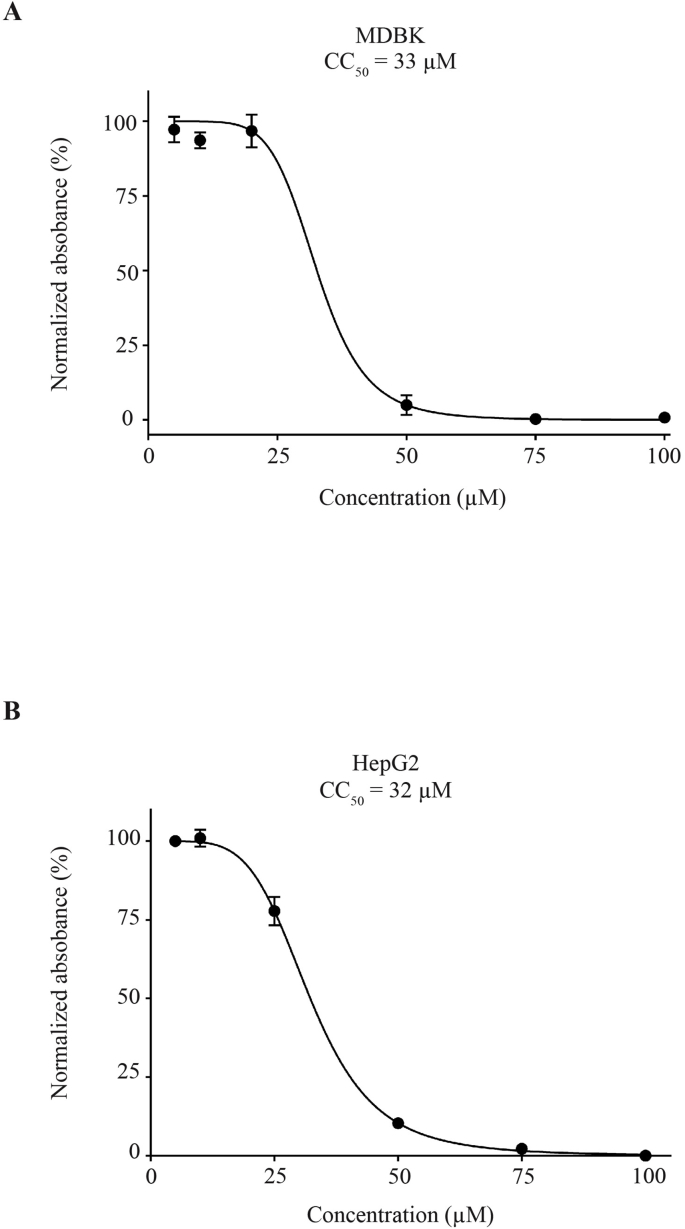


The mechanism of action (MOA) behind these commonly observed anthelmintic phenotypes (i.e. surface membrane defects) is currently unknown, but extrapolating findings from other systems implicates membrane disruption, lipase inhibition, mitochondrial dysfunction and cholesterol homeostasis alterations (Slamenova et al., 2004; Handa et al., 2013; Kathuria et al., 2014; Kuzu et al., 2014) as possibilities. While this study's scope was not designed to explore MOA in detail, we sought to indirectly explore this area by assessing the general cytotoxicity of 700015 on two representative mammalian cell lines, MDBK and HepG2 (Supplementary Figure 2). Using an MTT assay to detect mitochondrial NAD(P)H-dependent oxidoreductase activity, CC_50_ values calculated for 700015 were found to be similar for both bovine (CC_50_ = 33 μM) and human (CC_50_ = 32 μM) cell lines; these data indicated a low to moderate degree of general cytotoxicity for this triterpenoid. These findings are similar to those published previously, where moderate 700015 - mediated cytotoxicities on SK-OV-3 (human ovary malignant ascites; CC_50_ > 10 μM), HCT15 (human colon adenocarcinoma; CC_50_ > 10 μM), A549 (human non-small-cell lung adenocarcinoma; CC_50_ = 9.4 μM) and SK-MEL-2 (human skin melanoma; CC_50_ = 4.1 μM) cell lines were demonstrated (Kim et al., 2018). Nevertheless, when compared to the effect that 700015 had on the stages of helminth development studied herein for both species, the selectivity indices (SI = CC_50_/IC_50_) of this triterpenoid ranged between 3 and 46; the exception being adult liver fluke where the SI was 2 (Table 2). These values demonstrated that 700015 exhibited more selective activity against the parasitic flukes than the mammalian cell lines. Indeed, these SI values, generally, exceeded those obtained for related diterpenoids currently under study in our laboratory (Edwards et al., 2015; Crusco et al., 2018). Collectively, a window of selectivity between surrogate mammalian cell lines and flukes (in some cases, moderate to high) was established for 700015, suggesting that medicinal chemistry optimisation of this triterpenoid could lead to the generation of more specific anthelmintic analogues.

**Table 2:** Summary of anthelmintic activities and selectivity indices of 700015.

	Biological material and parameters assessed		EC_50_ (parasites) and CC_50_ (cells) values	Selectivity Index^b^
*F. hepatica*	Newly excysted juvenilles	Motility	2.4 μM	13.3
		Phenotype	2.4 μM	13.3
	Immature	Motility	0.7 μM^a^	45.7
	Adult	Motility	15.6 μM	2
*S. mansoni*	Schistosomula	Motility	1.9 μM	16.6
		Phenotype	2.6 μM	12.2
	Immature	Motility	3.4 μM	9.4
	Adult Male	Motility	7.4 μM	4.3
	Adult Female	Motility	10.3 μM	3.1
Cell Lines	MDBK	Viability	33 μM	
	HepG2	Viability	32 μM	

a The EC_50_ of 700015 on *F. hepatica* juveniles is an estimate only.

b Selectivity indices for *F. hepatica* lifecycle stages were calculated from MDBK CC_50_ values; selectivity indices for *S. mansoni* lifecycle stages were calculated from HepG2 CC_50_ values.

In summary, a novel phytochemical (700015) was isolated from the bark of *A. procera* and chemically defined as a triterpenoid containing a tetracyclic steroid-like nucleus and lactone side chain. These core structures were associated with moderately potent and selective anthelmintic activities against larval, immature and mature lifecycle stages of both *F. hepatica* and *S. mansoni* flatworm parasites. Further investigations of this novel triterpenoid for the control of both fasciolosis and schistosomiasis are warranted.

## Conflicts of interest

The authors indicate that they have a collaborative relationship with Bimeda Ltd, UK, which may have a direct or indirect financial interest in the subject matter discussed in the manuscript.

## Appendix A Supplementary data

Data Profile Data Profile
