# Supplementary material for: An Abies procera-derived tetracyclic triterpene containing a steroid-like nucleus core and a lactone side chain attenuates in vitro survival of both Fasciola hepatica and Schistosoma mansoni
Source: Int J Parasitol Drugs Drug Resist. 2018 Oct 26;8(3):465–74. doi: 10.1016/j.ijpddr.2018.10.009 (PMC6216039; doi:10.1016/j.ijpddr.2018.10.009)
Supplement: Supplementary Data Legends [file mmc2.docx]

**Supplementary Data**

**Supplementary Figure 1.** Structure and simplified molecular-input line-entry system (SMILE) of the ten triterpenoids isolated from *Abies* sp. and used in this study. LogP values of these triterpenoids are also indicated.

**Supplementary Figure 2.** Dose response titration of 700015 against both *Bos taurus* MDBK and *Homo sapiens* HepG2 cells.
