# Supplementary material for: An Abies procera-derived tetracyclic triterpene containing a steroid-like nucleus core and a lactone side chain attenuates in vitro survival of both Fasciola hepatica and Schistosoma mansoni
Source: Int J Parasitol Drugs Drug Resist. 2018 Oct 26;8(3):465–74. doi: 10.1016/j.ijpddr.2018.10.009 (PMC6216039; doi:10.1016/j.ijpddr.2018.10.009)
Supplement: Supplementary Fig. 1 [file mmc3.docx]

**Supplementary Figure 1**

| Code  (and LogP*) | Structure | SMILE |
| --- | --- | --- |
| 700015 (6.6) |  | O=C1CCC2(C)C(CC=C3C2CCC4(C)C3(C)CCC4C(C)CC5(O)OC(C(C)=C5)=O)C1(C)C |
| 700019  (5.8) |  | OC1CCC2(C)C(CC=C3C2CC=C4C3(C)CCC4C(C)CC(O)/C=C(C(O)=O)\C)C1(C)C |
| 700234  (6.8) |  | OC(C1(C)C)CCC2(C)C1=CCC3C2=CCC4(C)C3(C)CC5C4C(C)CC6(C=C(C)C(O6)=O)O5 |
| 700496  (-1.2) |  | CC1(CO)C(OC2OC(CO)C(O)C(O)C2OC3OC(C)C(O)C(O)C3O)CCC4(C)C1CCC5(C)C4CC=C6C5(CCC7(C(OC8OC(COC9C(O)C(O)C(OC%10OC(C)C(O)C(O)C%10O)C(CO)O9)C(O)C(O)C8O)=O)C6CC(C)(C)CC7)C |
| 700500  (-3.1) |  | CC1(CO)C(OC2OC(CO)C(O)C(O)C2OC3OC(C)C(O)C(OC4C(O)C(O)C(O)C(CO)O4)C3O)CCC5(C)C1CCC6(C)C5CC=C7C6(C)CCC8(C(OC9OC(COC%10C(O)C(O)C(OC%11OC(C)C(O)C(O)C%11O)C(CO)O%10)C(O)C(O)C9O)=O)C7CC(C)(C)CC8 |
| 700501  (-2.1) |  | CC1(CO)C(OC2OC(CO)C(O)C(O)C2OC3OC(C)C(O)C(OC4C(O)C(O)C(O)C(C)O4)C3O)CCC5(C)C1CCC6(C)C5CC=C7C6(C)CCC8(C(OC9OC(COC%10C(O)C(O)C(OC%11OC(C)C(O)C(O)C%11O)C(CO)O%10)C(O)C(O)C9O)=O)C7CC(C)(C)CC8 |
| 700502  (-0.5) |  | CC1(CO)C(OC2OC(CO)C(O)C(O)C2O)CCC3(C)C1CCC4(C)C3CC=C5C4(C)CCC6(C(OC7OC(COC8C(O)C(O)C(OC9OC(C)C(O)C(O)C9O)C(CO)O8)C(O)C(O)C7O)=O)C5CC(C)(C)CC6 |
| 700503  (-2.2) |  | CC1(CO)C(OC2OC(CO)C(O)C(O)C2OC3C(O)C(O)C(O)C(C)O3)CCC4(C)C1CCC5(C)C4CC=C6C5(C)CCC7(C(OC8OC(COC9C(O)C(O)C(OC%10OC(CO)C(O)C(O)C%10O)C(CO)O9)C(O)C(O)C8O)=O)C6CC(C)(C)CC7 |
| 700638  (8.5) |  | CC1(C)C(OC(/C=C/C2=CC(O)=C(O)C=C2)=O)CCC3(C)C1CCC4(C)C3CC=C5C4(C)CCC6(C(O)=O)C5CC(C)(C)CC6 |
| 700657  (2.7) |  | O=C(C1(C)C)C(O)=CC2C1=CCC(C(CC(O)C3C(O)(C)C(/C=C/C(O)(C)C)=O)(C)C3(C)C4)C2(C)C4=O |

*LogP calculated by molinspiration services (<http://www.molinspiration.com/cgi-bin/properties>)
